# The use or generation of biomedical data and existing medicines to discover and establish new treatments for patients with rare diseases – recommendations of the IRDiRC Data Mining and Repurposing Task Force

**DOI:** 10.1186/s13023-019-1193-3

**Published:** 2019-10-15

**Authors:** Noel T Southall, Madhusudan Natarajan, Lilian Pek Lian Lau, Anneliene Hechtelt Jonker, Benoît Deprez, Tim Guilliams, Lawrence Hunter, Carin MA Rademaker, Virginie Hivert, Diego Ardigò, on behalf of the IRDiRC Data Mining and Repurposing Task Force, David Cavalla, Christine Colvis, Benoît Deprez, Kristina Hettne, Tim Guilliams, Virginie Hivert, Peter-Bram ‘t-Hoen, Lawrence Hunter, Caroline Kant, Jeffrey Krischer, Frédéric Marin, Madhusudan Natarajan, Jordi Quintana, Carin MA Rademaker, Jane Reed, Noel Southall, Stylianos Tsigkos, Rick Thompson, Annemieke Aartsma-Rus

**Affiliations:** 1 National Center for Advancing Translational Sciences, National Institutes of Health, Bethesda, MD USA; 2 Rare Diseases Drug Discovery Unit, Takeda, Cambridge, MA USA; 3 IRDiRC Scientific Secretariat, Inserm-US14, Paris, France; 4 Université de Lille, Inserm, Institut Pasteur de Lille, U1177 – Drugs and Molecules for Living Systems, Lille, France; 5 Apteeus, Campus Institut Pasteur de Lille, Lille, France; 6 HealX, Cambridge, UK; 7 University of Colorado, Denver School of Medicine, Denver, USA; 8 University Medical Center Utrecht, Utrecht, the Netherlands; 9 EURORDIS-Rare Diseases Europe, Paris, France; 10 Chiesi Farmaceutici SpA, Parma, Italy

**Keywords:** Rare disease, Data mining, Repurposing, Repositioning, Therapies, Drug development, Orphan drug, Orphan medical product, IRDiRC

## Abstract

The number of available therapies for rare diseases remains low, as fewer than 6% of rare diseases have an approved treatment option. The International Rare Diseases Research Consortium (IRDiRC) set up the multi-stakeholder Data Mining and Repurposing (DMR) Task Force to examine the potential of applying biomedical data mining strategies to identify new opportunities to use existing pharmaceutical compounds in new ways and to accelerate the pace of drug development for rare disease patients. In reviewing past successes of data mining for drug repurposing, and planning for future biomedical research capacity, the DMR Task Force identified four strategic infrastructure investment areas to focus on in order to accelerate rare disease research productivity and drug development: (1) improving the capture and sharing of self-reported patient data, (2) better integration of existing research data, (3) increasing experimental testing capacity, and (4) sharing of rare disease research and development expertise. Additionally, the DMR Task Force also recommended a number of strategies to increase data mining and repurposing opportunities for rare diseases research as well as the development of individualized and precision medicine strategies.

## Introduction

An estimated 7000 rare diseases affect over 350 million people worldwide [1, 2]. As a guide, a disease is considered rare when it affects less than one in 2000–10,000 in a population [3–5]. About half of those affected by a rare disease are children, and 30% of them will not live to see their fifth birthday [2]. At present, fewer than 6% of rare diseases have an approved treatment option; this represents a vast unmet medical need and opportunity to provide new orphan drugs [6]. However, the development of orphan drugs face many challenges, including the often very limited understanding of disease epidemiology, manifestations, heterogeneity and progression; a lack of consensus on which patient-centered clinical endpoints to use; and a complicated clinical trial design and organization; all of which leads to a time and resource-consuming process [7]. The (sometimes extremely) limited size of the population identifiable and eligible to be enrolled in clinical studies also lends to greater uncertainties on efficacy (underpowered trials and limited evidence) as well as safety (limited safety population) at the time of marketing authorization. Just as with common drugs, the low success rates of drugs in clinical trials creates additional complications and costs to drug development [8].

To improve research and development as well as market and access conditions for orphan drugs, numerous dedicated regulations, policies, and incentives to encourage investment in orphan drug development have been introduced [3, 9]. While these measures have spurred drug development for rare diseases – nearly half of all new medicines approved in 2015–2018 were therapies for rare diseases, and the overall success rate for orphan drugs from Phase I to approval between 2006 and 2015 is about 25% [10] – a recent analysis shows that, from 2011 to 2018, only approximately 300 orphan drugs are available to treat 320 diseases in the USA and Europe combined (*Jonker AH* et al.*, manuscript in preparation*). The medical need far outstrips drug developers’ ability to deliver new therapies and there is an urgent need for new treatments for rare diseases.

It is perhaps surprising that therapeutic opportunities for rare diseases remain limited at a time when the capacity to generate data continues to grow. These unprecedentedly large amounts of data – from rare to non-rare to common diseases – have challenged researchers trying to make sense of it [11]. Meanwhile, data sharing initiatives also open up access to new types of data including patient records and other real-world data. These data are ripe for analyses using big data techniques, including computational models that unveil molecular mechanisms and similarities among clinical phenotypes, predict compound-ligand interactions, perform high-throughput screening of molecules against cell lines and network-based in silico drug efficacy screening, and data mining for potential therapeutic targets based on existing knowledge [12–14]. At the point of convergence of several academic research fields (e.g., applied mathematics, computer science, artificial intelligence, statistics and machine learning), data mining takes advantage of the potential to carry out novel multi-dimensional analytics to connect data on diseases, mechanisms, proteins, and drugs [15–17]. Pieced together, data mining methods enable the discovery of new or the repurposing of previously known pharmaceutical compounds in the development of treatments for new indications [18–20].

The process of drug repurposing, or drug repositioning, involves the identification of a novel clinical use for an existing drug, i.e. to treat disease(s) for which it was not originally intended [21, 22]. While efficacy, safety, and pharmacokinetics cannot systematically be transferred from one disease to another, the potential advantages of drug repurposing for rare diseases include shorter development times and reduced costs due to reduced safety and pharmacokinetics risks, smaller rare disease clinical trial sizes than those for common diseases, and the potential recovery of investment through the rescue of previously failed compounds [7]. However, product development also needs to be made fit-for-purpose, for example, if a drug is approved for adults and the new indication is a childhood disease, additional safety tests will be needed. Furthermore, drug developers may remain reluctant to invest in repurposing when the drug patent has expired and the current, existing economic incentives are deemed lacking or ill-suited for repurposed drug, in particular upstream development required to gather supporting data for the application of orphan designation. Several recent reviews on drug repurposing and the challenges of this approach have been published and provide an excellent introduction to the subject [17–23] as well as development of a database of both approved and failed drugs along with their indications to provide insight into past repurposing experiences [24]. Repurposing has been particularly valuable in rare diseases. A 2017 study reported that among the US FDA approvals were 73 drugs which served the mass market before repositioning into the orphan space and 83 drugs have been approved for multiple orphan indications; eight drugs appear under both these categories [25, 26].

The International Rare Diseases Research Consortium (IRDiRC) was launched in 2011 to stimulate and coordinate basic and clinical research, encouraging translational, preclinical and clinical research [27]. Among IRDiRC’s actions to support therapeutic development, it set up the multi-stakeholder Data Mining and Repurposing (DMR) Task Force to examine the potential locked within existing datasets and realized by the use of biomedical data mining to better understand rare disease pathophysiology, identify new repositioning opportunities for therapeutic intervention, and predict whether a drug will have a clinical therapeutic effect [1]. This manuscript provides an overview of the main outcomes and recommendations resulting from the work of the DMR Task Force – examining how to better support data mining efforts that can accelerate the development of therapies for rare diseases.

## Maximizing rare disease development requires strategic infrastructure investments

The DMR Task Force was convened through IRDiRC. Initially seeded from interested IRDiRC members including public and private research sponsors, biopharmaceutical companies and patient advocates, it was expanded to include researchers with significant data mining and drug repurposing experience. The DMR Task Force met regularly by phone and through an in-person workshop hosted by Dr. Jordi Quintana at the Parc Científic de Barcelona. The group reviewed current approaches and critically assessed case studies of data mining and drug repurposing programs to develop consensus recommendations that would stimulate future research efforts. The DMR Task Force identified four key success factors for realizing the full potential of our available data and accelerating rare disease research productivity, not only to the benefit of the above-mentioned but the entire ecosystem of rare disease drug development:

- Improving the capture and sharing of self-reported patient data
- Better integration of existing research data
- Increasing experimental testing capacity
- Sharing of rare disease research and development expertise

### Improving the capture and sharing of self-reported patient data: learning more from how patients have been treated in the past

Repurposing efforts start with an extensive understanding of the target disease, its pathophysiology and its clinical phenotype. However, for many rare diseases, clinical information is scarce and scattered. Investing in the capture and sharing of self-reported patient data, in addition to data obtained in a research setting, supports both the evolution of medical practice and the opportunity to repurpose existing products and/or develop new products.

Patients themselves are increasingly involved in reporting of their conditions and outcomes using social media portals (e.g., Facebook, health fora) and patient-centric research platforms (e.g., PatientsLikeMe, Genetic Alliance’s PEER, RareConnect, Web-Radr) [28–31]. However, such data collection can vary in quality and sophistication; some tools are better designed and fit-for-purpose than others. Variable data standards, privacy concerns, data ownership silos as well as non-robust and/or inaccurate reporting all add to the complexity of using these data. Nevertheless, patient engagement in the research and drug development cycle is increasingly implemented and carries significant weight for the regulatory review process [32, 33]. Patient advocacy organizations can play a role in this process. The aggregation of data should be done in a non-conflicting or non-competitive manner.

Data standards are critical to understanding and using data. The DMR Task Force identified a significant opportunity in collecting clinical data compliant with regulatory standards to ensure maximal utility in both research and for future product development. Standardized collection qualified to fit with regulatory standards, created in consultation with patients and clinicians, and developed by IT providers – ideally validated for regulatory use – should be a widely-adopted multi-stakeholder approach [34, 35]. There are similar opportunities for industry and patient communities to collaborate on reporting standards and further incentivize sponsors that benefit from datasets made available by patients to contribute their own proprietary data back to the community. There is a need for data brokers who can aggregate and code data to enable development or enhancement of clinical practice, and this set of expertise feeds back to the improvement of data mining methods. The development of the Rare Disease Clinical Research Networks in the US and of the European Reference Networks for rare diseases in the EU both serve as models to help address these issues [36, 37].

In addition to data in support of clinical phenotype understanding, one additional significant gap in patient’s data collection that can boost repurposing is on the off-label use of medicines, including experience, mode of use, posology, efficacy, and side effects. Despite its widespread practice, off-label prescription and outcome data are difficult to collect for several reasons, including administrative ones such as the miscoding of diagnoses to enable reimbursements. Meanwhile, patients are increasingly reporting their treatment histories using online platforms, creating patient-reported source of information on off-label use in real time [38]. Attempts have been made on automated detection of off-label drug use based on data mining approach, which could lead to a better collection of real-world data of medicine use [39]. The creation of a database of off-label use of medicinal products was proposed in the STAMP reflection paper, and the recently launched European Reference Networks for Rare Diseases should be taken advantage of to perform better data collection and alignment of healthcare data in clinical settings [37, 40]. An additional opportunity to gather off-label use data arise where physicians contribute real world data to post-marketing surveillance by reporting back in return for authorization or conditional authorization. Similarly, researchers funded for studies of off-label uses should be required to share their data. Such systematic captures can facilitate the identification of new repurposing opportunities and support the analysis of benefits and risks conferred by a treatment to the patients.

### Better integration of existing research data: generating disease dashboards to coordinate rare disease research

The rare disease community cannot work in data silos. Sharing of information and samples will enhance disease understanding, help generate new hypotheses, and facilitate drug development via repurposing. Contextual knowledge of diseases contributes to the development of the right strategy to generate experiments, animal models, and approaches to validate pre-clinical and clinical studies. Data sharing also allows the identification of knowledge gaps, thus points where to invest in research.

The DMR Task Force recommends supporting the development of a “Target in Disease” dashboard to contain data relevant to a disease and potential interventions that can highlight, among others, where knowledge gaps exist and which missing data would increase the value of programs. This pre-competitive tool maximizes data mining and computational modelling capacity and should include answers to common questions that support research prioritization (e.g., mechanistic hypothesis, pharmacology against known targets, maximum exposure based on safety finding, reference data sources) and be made available to all. Ideally, this resource would be an integrated, curated disease map that explores pathway perturbation, connects to broader indications, enables analyses of research and clinical data to generate putative targets, and links to information in the literature analyzed using data mining. Data mining efforts will lend itself to an integrated, structured disease dashboard with the capability to systematically assess the strengths and weaknesses of new clinical hypotheses and prioritize further work. Subsequently, clinical judgement and individual assessments of human experts on different aspects of clinical plausibility, consideration of uncertainty, and level of evidence should be carried out to establish the robustness of the hypotheses and development priorities; misuse or misinterpretation of machine generated dashboard data may lead to wasted efforts.

The true value of public knowledge is in its curation and integration. For rare diseases, it is strongly opined that this should be conducted in an open source manner and contribute to the building of such knowledge. This knowledge, in return, should remain open for access by the community to incentivize further collaboration among stakeholders to maximize the potential application of the tool and data generated. However, methodology development and curation involve a significant amount of work, while the creation and maintenance of pre-competitive, open source databases requires financial support. The current system of licensing agreements hinders the re-use of data and the publication of derivative data, even in the case of public data, resulting in barriers to data access in the short term and undermining the sustainability of the data source in the long term. The DMR Task Force strongly recommends Creative Commons or equivalent licensing that allows data integration and facilitates mining but acknowledge that a sustainable funding model is needed as a first step to resolving this issue.

Furthermore, in building the disease dashboard, the adherence to FAIR – findable, accessible, interoperable and reusable – guiding principles is vital [41]. Moreover, the community data sharing practices should ideally be extended to ensure that data provenance information is always available, access is managed in accordance to consent, appropriate coding of disease pathophysiological information is done to facilitate machine readability, and datasets are interoperable. Several disease dashboards initiatives and tools for data organization and sharing in line with the FAIR data principles exist (e.g., Open PHACTS, Monarch Initiative, tranSMART, RD-Connect, Automatable Discovery and Access Matrix) [42–46] and upon which the community can, and should, build on. Not only will the effort of building further on existing FAIR dashboard initiatives maximize the utility of these infrastructures, it may contribute towards their sustainability. A Charter could be built that applies across these FAIR initiatives and drives the movement for a common approach so that public knowledge can converge and be made available for use. The Charter should also set out common minimum data sharing expectations when collaborating with commercial providers to develop rare disease knowledge. As the FAIR principles do not enforce the implementation of specific technologies, standards or ontologies, it would be wise to reach consensus on a reference implementation for the field of drug repurposing in rare diseases.

One important point of leverage may lie in linking rare diseases to common diseases. Rare diseases can provide unique insights into the underlying causes, potential therapeutic approaches and personalized medicines for common diseases, and collaborating with existing resources to do so would potentially provide wide-ranging benefits.

### Increasing experimental testing capacity: validating new repurposing hypotheses and data mining approaches

The experimental and clinical testing capacity to accelerate rare diseases therapeutic development must be increased. At each stage of development, some project attrition occurs simply due to a lack of testing capacity [47]. For the field of data mining in particular, this issue is critical, hampering the evaluation and development of new approaches. The ability to create cell or animal models bearing the exact mutations found in the genome of patients is of paramount importance to validate candidate molecules generated in silico or to screen collections of approved drugs in an unbiased manner, prior to orphan designation status applications. Such models, tailored with specific molecular defect (often point mutations) can now be obtained from patient biopsies, or produced with state-of-the-art genome editing technologies that should be made easily available to researchers via shared platforms. In evaluating orphan designation applications, as the generation of a relevant animal model may take years yet only reflected in the end a small fraction of a given orphan disease, a greater weight on data obtained on cell models derived from patient cells should be considered by regulatory authorities.

A shared capacity for experimental validation may help to overcome this barrier. Community assessment is one specific model to catalyze the evaluation of computational methods and is highly recommended to bridge the cultural gap between computational and experimental researchers thus to obtain fair assessment of each other’s research approaches. At the NIH/NCATS, initiatives have been set out to create such collaborative research by offering funding to validate in silico strategies with pre-clinical testing [48]. One key incentive of this and other experimental testing programs is that intellectual property is protected during the testing process. Without this, the commercial incentive for further development becomes limited. More joint collaborative efforts should be encouraged by IRDiRC and other research structures, be it through joint (transnational) funding calls or through specific criteria requirement (e.g., for an Innovative Medicines Initiative project, the support of industry partners is needed). In particular, Public-Private partnership is of high added-value in the field of repurposing.

Current clinical validation capacity is also limited. While some publicly-funded programs are available to facilitate clinical development (e.g., NIH’s Therapeutics for Rare and Neglected Diseases program and FDA’s Office of Orphan Products clinical grant program), complementary funding instruments must be developed. Additionally, determination of the collaborative approach in running these types of clinical trials is also needed.

### Nurturing rare disease research and development expertise: sharing expertise to make the best use of limited resources

The development of rare disease therapies presents its own unique set of challenges throughout the discovery and development process. To ensure success, attracting and training talented researchers with the right skill sets and understanding of the financial, clinical, and regulatory aspects of product development is essential. Education regarding various aspects across the drug development spectrum and different business model approaches will help to address issues such as the drug development process; how to obtain funding support; when to ask for regulatory advice; what are the regulatory protections or support available for patented molecules, marketing authorization, and orphan designation; compassionate use and authorization guidelines; data requirement to improve and/or complete a (pre-clinical) data package; caution in using pre-existing safety data when repurposing; and peer-to-peer collaboration models. Researchers would also have a better chance of achieving a commercial development partnership if more specific information is provided when approaching companies, including the targeted rare disease, its mechanism of action, the bioavailability, the duration of treatment, patient-centric endpoints, and the end goal.

The DMR Task Force emphasized the importance to provide not only written educational content but to also support in-person development boot camps, workshops and summer schools (e.g., EURORDIS Open Academy and Summer School [49]) to address specific circumstances of a project’s development.

The DMR Task Force also explored sharing rare disease drug development expertise on a pro bono basis. Many professional groups expect members to make some charitable contribution of their time, paying forward the help they received as part of their own training, through clinics or by acting in a review capacity. IRDiRC should facilitate access to accredited experts whom researchers could interact with given specific question(s) they need answer(s) to. This is particularly important given there are few established rare diseases drug development paths to follow, and sponsors need to be clever in attracting commercial interest – even more so in the case of repurposing.

## Increasing data mining and repurposing opportunities

It is undisputed that biomedical researchers have an incredible capacity to generate large datasets, while data sharing is opening access to exciting datasets that provide meaningful context and depth to analyses. Novel analytic models are increasingly being harnessed to enhance researchers’ ability to make new discoveries from mined data, better understand rare diseases and identify new therapeutic opportunities (see Table 1). Continued advances in this line of research depend on several factors, many of which are outlined above. First, the productive integration of biomedical research (patient-derived) data by fostering adherence to data standards and FAIR data principles must be enabled. Second, supporting the broad sharing of research datasets, especially clinical datasets, is critical. Third, the validation and improvement of data mining techniques and approaches requires robust interactions among researchers of multi-disciplinary background, including access to experimental testing resources and through community assessments. Market incentives currently encourage data mining companies with promising algorithms to either move into tight collaboration with pharmaceutical companies which may limit the scope of assets interrogated or to adapt the algorithms for use in different domains with more commercial potential (e.g., sales and marketing, with more data available for validation). However, a case can also be made for the ability of the developers to retain the algorithm or even collaborate in the open source space in order to impart an opportunity of further development and continued utility. Nonetheless, it is becoming increasingly important to incentivize algorithm developers to remain in the medical research space, in part through removal of barriers to validation. Finally, developing this specialized type of research capacity will also require a dedicated training and recruitment effort. With these investments, biomedical researchers are positioned to significantly accelerate the development of new therapies for rare diseases over the next decade.

**Table 1:** Examples of data mining approaches for the identification of drug candidates for new diseases starting from libraries of approved pharmaceuticals

Case 1: Repurposing drugs for Fragile X Syndrome	
Healx, in partnership with FRAXA Research Foundation, employs machine learning algorithms and computational biology as the basis of its in silico Disease-Gene Expression Matching (DGEM) pipeline – with subsequent pharmacological expert reviews - to identify drug-disease connections. This approach results in several candidates, of which three were tested in in vivo mice studies, and the most promising chosen to progress through Phase IIa trials. It took only 15 months from project initiation to readiness for clinical trial [50, 51].	
Case 2: Tolcapone repurposed to treat transthyretin amyloidosis (ATTR)	
Following the use of a proprietary virtual screening platform, SOM Biotech identified tolcapone (SOM0226, or CRX-1008) as potential treatment for ATTR, a rare genetic degenerative disease where abnormal build-up of amyloid takes place and deposited in different organs and tissues, notably the nervous system and myocardium. Clinical validations with Phase II trials led to the granting of orphan drug designation by the FDA for all types of ATTR. The knowledge gained was leveraged against the execution of a licensing agreement for the clinical development and commercialization of this repurposed drug, an oral medication used as adjunct in the treatment of Parkinson’s disease [52, 53].	
Case 3: TEE886 in pseudo-adrenoleukodystrophy	
APTEEUS, through its ID2STOP Orphan (Individualized Drug Selection Technology for Orphan Patients) program, gathered over 1500 marketed drugs to build a pharmacopeia which can be systematically screened against a patient’s cells which bear the causative effect of the disease. Once potential drug candidates were identified, functional assays were carried out to determine efficacy and potency in rescuing the disease phenotype. In a case of a pseudo-adrenoleukodystrophy patient, incubation of his skin fibroblasts with TEE886 shows restoration of the profile of very long chain fatty acids which would otherwise accumulate in all cells in his body (*Conference communication*, [54]).	
Case 4: Fluspirilene as candidate anti-cancer drug	
The cyclin-dependent kinase 2 (CDK2) is an attractive anti-cancer drug target given its roles in controlling cell proliferation. A group of academic researchers created a free, open-source protein-ligand docking software to conduct in silico screening of FDA-approved small molecular drugs against CDK2. Nine compounds were subsequently tested in vitro of which the anti-psychotic drug fluspirilene was identified as a potential CDK2 inhibitor. Further in vivo mice studies show the potential of the repurposing of fluspirilene as anti-hepatocellular carcinoma [55].	

Drug repurposing is a viable strategy to counter the decreasing productivity gaps of the traditional drug development model and there are a number of ways to increase new repurposing opportunities. First, the surveillance of existing medical practice and off-label use must be improved to increase reporting of findings that will accelerate the discovery of new uses. Second, complementary systematic screening and data mining approaches show exciting potential to fully leverage recent exponential increases in data and knowledge about biology and human health. Nevertheless, experimental validation of repurposing candidates, both in model systems and clinically, remains a significant bottleneck to the development of repurposing opportunities and their enabling technologies. Intellectual property and other strategic protections are essential to attract and protect the capital investment that clinical development requires.

Developers bringing repositioned products to market use a variety of approaches to protect their investments. In many cases, commercial development is feasible and continued innovation in this area is expected. Approaches include patenting novel uses of existing medicines or molecules; developing new dose, formulation or route of administration; reformulating new, specialized products with existing active ingredients; combining multiple molecules to form a novel drug; developing new chemical entity based on clinical proof-of-concept of a known molecule; protecting the way a product is prescribed; securing system of control by regulators to prevent substitution of products with generics; and develop unique, patient-focused commercialization strategies.

In cases where commercial development is not possible, public funders and clinical researchers, together with other rare disease stakeholders, need to work together to deliver therapeutic opportunities for patients [56]. This include gradual evolution of medical practice without the creation of new medicinal products, through better understanding of a disease’s natural history and comorbidity, and small changes in treatment protocols can provide significant benefit to patients. Additionally, the role of patients and their advocates in drug discovery and development must not be underestimated, from fundraising to driving clinical trials [57].

## Conclusion and perspectives

With so few rare diseases having an approved treatment option, patients need the research community to consider all possible avenues for discovery, especially the re-use of existing drugs and data to help tackle this vast unmet medical need. Already, rare diseases research has fundamentally changed what we think of as a medicine, producing notable innovations including the first enzyme replacement therapy (Ceredase® in 1991), first antisense oligonucleotide therapy (Vitravene® in 1998), first targeted cancer therapy (Gleevec®/Glivec® in 2001), first in-vivo gene therapy (Glybera® in 2012), first stem cell-based medicinal product (Holoclar® in 2015), and first ex-vivo gene therapy (Strimvelis®, 2016). With computational advances and data sharing capability, new therapeutic opportunities abound and have the potential to bring forth drug discovery and repurposing. According to a market research analysis by Business Communications Company Research, the global market for drug repositioning will grow to over $31 billion by 2020, up from about $24 billion in 2015, thus representing large commercial possibility [58]. A presentation by the FDA in September 2013 showed about half of the orphan drugs it approved were repurposed while an analysis published in January 2017 showed the overall figure to hover around one-third [59]. The medical community has yet to realize the full potential of the drugs and compounds that are already available. Improving the capacity to capture serendipitous findings and developing new methods to systematically identify these opportunities, thus repositioning these products as new treatments to the patients that are still waiting. Moreover, these strategies are applicable not only to rare diseases, but poised to show the way forward for individualized and precision medicines.

## Data Availability

Not applicable

## Abbreviations

DMR: Data mining and repurposing
EMA: European Medicines Agency
FAIR: Findable, Accessible, Interoperable, Reusable
FDA: Food and Drug Administration
IRDiRC: International Rare Diseases Research Consortium
